# Sleep, Caffeine, BMI, and Pressure Pain Threshold in Temporomandibular Disorder Patients: An Observational Study

**DOI:** 10.7759/cureus.57703

**Published:** 2024-04-06

**Authors:** May W Al-Khudhairy, Ghadah Bandar Alkhamsi Alqahtani, Abeer Mohammad A Altwijri, Reem Abdullah Aladwani, Daad Hosam AlYousof, Luluh Nasser AlNajdi, Ghassan Al-Turki

**Affiliations:** 1 Oral Biology, College of Medicine and Dentistry, Riyadh Elm University, Riyadh, SAU; 2 Dentistry, College of Medicine and Dentistry, Riyadh Elm University, Riyadh, SAU; 3 Orthodontics, Faculty of Dentistry, King Abdulaziz University, Jeddah, SAU

**Keywords:** temporomandibular disorder, pain pressure threshold, females, (ppt) pain pressure threshold, (tmd) temporomandibular disorder, sleep, caffeine

## Abstract

Background: Temporomandibular disorders (TMDs) represent a multifactorial condition involving a multitude of symptoms of the temporomandibular joint that emanates a series of distress. Understanding the relationship between these lifestyle factors and pain perception in TMD patients is essential for optimizing their management and care. This study delves into the intricate interplay between sleep, caffeine consumption, body mass index (BMI), and the potential effect on pressure pain threshold (PPT) values among individuals with TMDs.

Materials and methods: This is an observational study. Data were collected from a convenient sample of female patients at a single center in Riyadh city, between the ages of 20 and 50 years. The variables collected were based on an operator-designed questionnaire, the symptom questionnaire, and the Diagnostic Criteria for Temporomandibular Joint Disorders (DC/TMD).

Results: A total of 139 participants were included in the study, appraising the occurrence of TMD and pain as per reports of caffeine intake and sleep duration. The observed outcomes indicate that the amount of sleep has a significant effect on the PPT values in TMD patients. This study highlights the substantial impact of sleep duration on lowering PPT values in individuals with TMDs. The findings highlight the importance of considering sleep duration and caffeine intake in the comprehensive management of TMD patients. There was no effect of BMI on this particular sample.

Conclusion: This study shows a positive correlation between sleep and pain and TMD, caffeine, and pain. A deeper understanding of these relationships could pave the way for more effective pain management strategies and personalized treatment approaches tailored to the unique needs of TMD patients. BMI had no effect.

## Introduction

Sleep is a vital component of overall health, with insufficient rest causing a pervasive sense of fatigue, impacting work and daily activities, and posing safety risks due to slowed reflexes [1]. A wide array of factors contribute to prevalent sleep deficiencies, including hectic lifestyles, social night activities, shift work, and reliance on stimulants like caffeine, which all negatively affect health and contribute to sleep deficits, going against the recommended ≥ seven hours of sleep for adults aged 18-60 years. There is a global interest in caffeine's impact on health, with studies showing it can exacerbate anxiety at various intake levels, thereby confirming its potent effects [2]. Caffeine, a commonly consumed psychoactive substance, has a complex relationship with sleep and is capable of producing both beneficial and detrimental effects, which calls for further examination. Coffee causes sleep disturbances primarily through its main constituent, caffeine, which acts as a central nervous system stimulant. By blocking adenosine receptors, caffeine delays sleep onset, reduces total sleep time, and disrupts sleep architecture [3-5]. For healthy adults, the generally accepted safe daily caffeine limit is 400 mg, or around three cups of coffee, which is not typically linked with negative outcomes like toxicity or dependence. However, exceeding 500-600 mg can lead to significant health concerns. Caffeine content varies across beverages, with soft drinks, teas, and coffee containing different amounts [3]. Regarding body mass index (BMI) and its influence on temporomandibular disorders (TMDs), a Korean national study noted no significant difference in TMD prevalence among males across BMI levels while a low BMI in females was significantly linked with TMD [4].

A cross-sectional study accounting for age, gender, migraines, depression, and sleep disorders found no significant BMI and painful TMD relationship, a link that vanished after adjusting for these variables [5]. Contrarily, another study identified a correlation between TMD and BMI in females, observing a BMI reduction after two years in those with TMD [6]. Myofascial pain (MFP) is a major health problem that often leads to chronic pain and is commonly seen in TMD patients. Trigger points within the masticatory muscles, detectable through palpation, contribute to MFP and can be categorized as latent or active based on the elicitation of pain. MFP can mimic other pain conditions, such as fibromyalgia, and is frequently a comorbid condition [7,8]. Pressure pain threshold (PPT), the minimum pressure causing pain, can be measured and is referenced by the Diagnostic Criteria for Temporomandibular Joint Disorders (DC/TMD) for standardization [9]. Notably, PPT tends to decrease with sleep deprivation, emphasizing the need for adequate sleep to avoid misdiagnosis in DC/TMD evaluations, suggesting that inadequate sleep can lead to reduced PPT [10,11]. Therefore, the aim of this study is to explore the dynamic between sleep, caffeine intake, and BMI in relation to PPT in female patients suffering from TMDs. By examining these factors, we seek to determine how lifestyle influences pain perception and to assess the significance of these variables in the management and treatment of TMD. The hypothesis formulated for our study posited that sleep duration would have a significant effect on PPT values among individuals with TMDs.

## Materials and methods

Study design and sample size estimation

The sample was collected from female residents who were living in Riyadh and seeking regular dental treatment at Riyadh Elm University's An Namudhajiyah campus. The examination was conducted on a convenient sample of 139 women between the ages of 20 and 50 years from the 1st of September until the end of November 2019, based on guidelines similar to another study [12]. The convenient sampling method was chosen for its efficiency in accessing our specific population within a limited timeframe and logistical constraints. Moreover, this approach aligned with our study's focused objectives on exploring targeted factors among female Riyadh residents seeking dental treatment for TMD.

Study registration

The protocol for this investigation, after a review of all ethical requirements outlined in the Declaration of Helsinki for scientific research, was approved by the Institutional Review Board (IRB) at Riyadh Elm University (REU) and assigned the approval number RC/IRB/2019/241.

Inclusion and exclusion criteria

Inclusion criteria included healthy female volunteers between the ages of 20 and 50 years attending dental university clinics. Subjects experiencing TMD-associated pain within the last 30 days were further considered for inclusion. The exclusion criteria comprised individuals with odontogenic and non-odontogenic pain, systemic disorders, greater than two missing premolars and/or molars (with the exclusion of wisdom teeth), removable intraoral hardware, brackets for orthodontic purposes, and ongoing prescription and over-the-counter medication for depression, headache, infection, and pain. Each prospective candidate was also considered case-by-case when occlusion was a concern.

Clinical examination

All potential participants were given the free choice to participate after the procedures were verbally explained. A paper-based consent form detailing the research presented for their approval or rejection was followed by an e-signature informed consent form in the Dentoplus system of the university (an electronic database). The DC/TMD were utilized for the participant examination [13]. Inter- and intra-examiners were five trained dental undergraduates, and the diagnosis was done by an experienced specialist. The personal and demographic details of the assessed participants were registered to hypothesize whether they could alter the pain threshold or not. The incisal relationships were measured by a UNC15 periodontal probe. Opening, lateral, and protrusive movements were measured by a dental caliper. Other information was obtained by either observation or history. Muscle and temporomandibular joint (TMJ) palpation was done with the aid of a manual algometer (Baseline Dolorimeters, Fabrication Enterprises Inc., White Plains, NY) with a flush round probe’s tip (1.52 cm^2^) that was directed toward the participant's muscle or joint. The pressure was consistently applied, and the gauge reading was calibrated until the pain was reported by the participant, which is their pain threshold. This is calibrated as 2 lbs for the muscles of mastication as per DC/TMD and 1 lb for the TMJ, respectively (unless the participant feels pain before this gauge reading of 2 lbs and 1 lb, respectively). The study employed a sample of conveniently selected female participants receiving treatment at REU dental clinics. Data collection encompassed the utilization of the DC/TMD questionnaire, complemented by manual algometry, a digital scale, and stethoscope examinations. The participants' height, weight, BMI, and daily caffeine consumption were recorded. Pain location and type experienced in the last 30 days were documented. Pearson correlation coefficients to assess relationships between variables were calculated to explore the potential correlation and impact of sleep quality, BMI, and caffeine intake on PPT values among TMD patients.

Study setting and assessed parameters

The examination was conducted in a dental clinic where the patient was in an upright position on the dental chair with the examiner stabilizing the patient's head passively and one layer of double-folded (4 x 4 cm) gauze placed over the pressure application area prior to the assessment of the PPT. Participants were directed to verbally express whenever they felt pain; according to their report, a consensus was reached by the examiner as to whether the response was in fact pain or pressure. The anterior, middle, and posterior bellies of the temporalis and the masseters were evaluated bilaterally while the participant was seated in an upright position [14]. One examiner assessed each area three times, and the average was recorded. TMJ noises were confirmed by a stethoscope (3M Littmann Classic II, Saint Paul, MN) [14].

## Results

**Figure 1:**
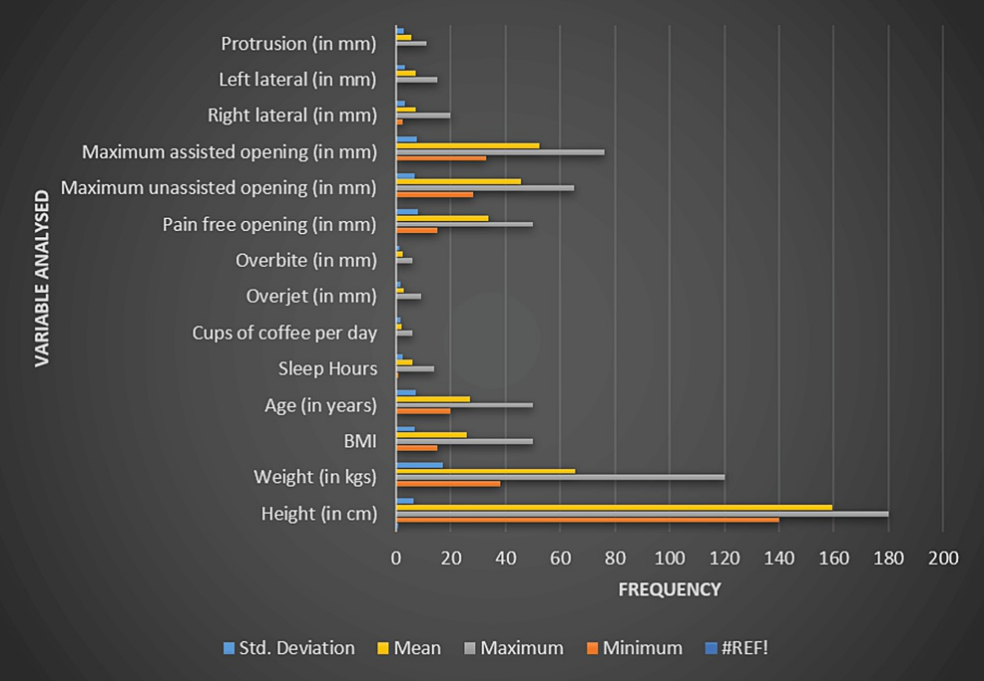
Baseline data of the included participants

The various physical and lifestyle parameters that were analyzed among the 139 participants included in the study are represented in Figure 1. The height of participants ranged from 140 to 180 cm, with an average of approximately 159.42 cm and a standard deviation of 6.57 cm. The weight varied between 38 and 120 kg, with a mean weight of around 65.33 kg and a standard deviation of 17.05. BMI figures spanned from 15 to 50, with a mean of 25.89 and a standard deviation of 6.63. The age of the subjects was between 20 and 50 years, with an average age of 27 years and a standard deviation of 7.03. Participants reported sleeping between one and 14 hours, with an average of roughly 5.87 hours and a standard deviation of 2.28. The number of cups of coffee consumed each day ranged from none to six, with an average intake of about 2.06 cups and a standard deviation of 1.53. In terms of dental measurements, overjet ranged from 0 to 9, with a mean of 2.69 and a standard deviation of 1.49, while overbite ranged from 0 to 6, with a mean of 2.24 and a standard deviation of 1.38. The pain-free opening ranged from 15 to 50, with an average of 33.62 and a standard deviation of 8.13; the maximum unassisted opening ranged from 28 to 65, with a mean of 45.51 and a standard deviation of 6.94; and the maximum assisted opening varied from 33 to 76, with a mean of 52.17 and a standard deviation of 7.59. Right lateral movement ranged from 2.5 to 20, with a mean of 7.30 and a standard deviation of 3.25; left lateral movement ranged from 0 to 15, with an average of 7.07 and a standard deviation of 3.20; and protrusion varied from 0 to 11, with a mean of 5.64 and a standard deviation of 2.71.

Cross-tabulation analysis with cups of coffee and various pain locations over the last 30 days indicated no significant correlation, with p-values of 0.954 for the location of pain, 0.662 for the location of side pain, 0.982 for the location of headache, and 0.909 for the location of side headache (Table 1). Additionally, the opening pattern (straight, corrected, or uncorrected) and the presence of pain disorders showed no significant correlation with coffee consumption, with p-values of 0.128 and 0.411, respectively. Myalgia, myofascial pain with referral, and arthralgia also did not show a significant correlation, with p-values of 0.790, 0.989, and 0.605. Lastly, the presence of headaches attributed to TMD and coffee intake did not demonstrate a significant correlation, with a p-value of 0.218.

**Table 1:** Cross-tabulation between cups of coffee and other variables TMD: temporomandibular disorder; TMJ: temporomandibular joint.

Variable analyzed	Category	Cups of coffee consumed per day							P-value
		0 cups	1 cup	2 cups	3 cups	4 cups	5 cups	6 cups	
Location of pain, last 30 days	None	73%	64%	75%	65%	75%	56%	40%	0.954
	Temporalis	15%	19%	13%	22%	25%	22%	40%	
	Masseter	7%	6%	6%	4%	0%	0%	0%	
	TMJ	0%	2%	3%	4%	0%	0%	0%	
	Other	0%	9%	3%	4%	0%	22%	20%	
Location of side pain, last 30 days	None	67%	64%	75%	65%	75%	56%	40%	0.662
	Unilateral	0%	9%	9%	9%	13%	0%	0%	
	Bilateral	33%	28%	16%	26%	13%	44%	60%	
Location of headache, last 30 days	None	40%	32%	19%	35%	38%	22%	40%	0.982
	Temporal	40%	51%	59%	48%	50%	56%	40%	
	Other	20%	17%	22%	17%	13%	22%	20%	
Location of side headache, last 30 days	None	47%	34%	19%	30%	38%	22%	40%	0.909
	Unilateral	7%	21%	22%	22%	13%	22%	20%	
	Bilateral	47%	45%	59%	48%	50%	56%	40%	
Opening pattern	Straight	33%	40%	25%	35%	63%	56%	40%	0.128
	Corrected	60%	53%	72%	57%	38%	44%	20%	
	Uncorrected	7%	6%	3%	9%	0%	0%	40%	
Pain disorders	None	87%	57%	59%	57%	50%	78%	60%	0.411
	Yes	13%	43%	41%	43%	50%	22%	40%	
Myalgia	None	67%	70%	63%	65%	75%	44%	80%	0.79
	Yes	33%	30%	38%	35%	25%	56%	20%	
Myofascial pain with referral	None	80%	83%	88%	87%	88%	89%	80%	0.989
	Yes	20%	17%	13%	13%	13%	11%	20%	
Arthralgia	None	80%	89%	78%	91%	88%	89%	80%	0.605
	Unilateral	20%	6%	16%	9%	0%	0%	20%	
	Bilateral	0%	4%	6%	0%	13%	11%	0%	
Headache attributed to TMD	None	60%	79%	72%	91%	88%	56%	80%	0.218
	Yes	40%	21%	28%	9%	13%	44%	20%	

When considering the location of pain experienced in the last 30 days, 50% to 100% of participants with no pain reported sleeping for different durations, ranging from one to 12 hours, with the highest percentages (100%) occurring at 11, 12, and 14 hours of sleep (Table 2). The temporalis muscle was affected in varying degrees, with 50% of those who slept for one hour experiencing pain, decreasing significantly to 0% for those who slept for 11 and 12 hours. Pain in the masseter muscle, TMJ, and other locations showed low percentages across different sleep durations. The p-value for the relationship between sleep hours and the location of pain was 0.499, indicating no statistically significant correlation. Regarding the location of side pain in the last 30 days, 50% to 100% of those with no side pain slept for various lengths of time, with 100% reported at 11, 12, and 14 hours. Unilateral and bilateral side pain was reported less frequently, with bilateral pain seen more in those who slept for four, five, and nine hours. The p-value was 0.423, suggesting no significant association between sleep duration and side pain. For the location of headaches in the last 30 days, participants with no headaches accounted for 25% to 67% across different sleep durations, with no headaches reported at 11 and 12 hours. Temporal headaches were more prevalent, with the highest frequency (75%) among those who slept for one hour. The occurrence of other types of headaches varied, with a p-value of 0.056, which indicates there may be a trend toward significance but is not statistically conclusive. The pattern of headaches on the side experienced in the last 30 days showed similar trends, with no side headaches being most common among participants who slept for 11 hours (67%). Participants experiencing unilateral and bilateral side headaches displayed varied percentages across the sleep hours, with bilateral side headaches being the most prevalent (77%) in those who slept for four hours. The p-value was 0.060, suggesting a potential trend but not a statistically significant one. The opening pattern of the jaw was categorized as straight, corrected, and uncorrected. Participants with a straight opening pattern constituted 25% to 100% across different sleep durations, with the majority (100%) sleeping for 12 and 14 hours. Those with a corrected opening pattern were most frequent at nine and 10 hours of sleep (80% and 75%, respectively), while uncorrected opening patterns were the least common and showed no clear relationship with sleep duration. The p-value was 0.607, indicating no significant correlation. Participants without pain disorders were mostly among those who slept for nine hours (100%), while those with pain disorders were highest among those who slept for 10 hours (75%). The p-value for this relationship was 0.466, which shows no significant correlation. For myalgia and myofascial pain with referral, the percentages of those without these conditions were generally high across all sleep durations, particularly at nine and 11 hours for myalgia and at one, four, and seven through 14 hours for myofascial pain with referral. The p-values were 0.858 and 0.277, respectively, both indicating no significant correlations. Arthralgia cases were examined, and it was found that those without the condition constituted the majority across most sleep durations, particularly at two hours (100%) and nine hours (80%). The p-value was 0.435, which again indicates no significant correlation. When looking at headaches attributed to TMD, those without such headaches were most numerous at two hours (100%) and nine hours (80%) of sleep. In contrast, those with TMD-related headaches were most frequent at one hour (75%) of sleep. The p-value here was 0.103, which does not denote a statistically significant correlation.

**Table 2:** Cross-tabulation between sleep hours and other variables TMD: temporomandibular disorder; TMJ: temporomandibular joint.

Item	Category	Number of hours slept													P-value
		1 hour	2 hours	3 hours	4 hours	5 hours	6 hours	7 hours	8 hours	9 hours	10 hours	11 hours	12 hours	14 hours	
Location of pain, last 30 days	None	50%	67%	62%	62%	52%	78%	75%	88%	40%	50%	100%	100%	100%	0.499
	Temporalis	50%	0%	8%	15%	30%	13%	20%	13%	20%	50%	0%	0%	0%	
	Masseter	0%	0%	8%	8%	6%	4%	5%	0%	20%	0%	0%	0%	0%	
	TMJ	0%	33%	8%	0%	0%	4%	0%	0%	0%	0%	0%	0%	0%	
	Other	0%	0%	15%	15%	12%	0%	0%	0%	20%	0%	0%	0%	0%	
Location of side pain, last 30 days	None	50%	67%	62%	62%	52%	74%	75%	88%	40%	50%	100%	100%	100%	0.423
	Unilateral	0%	33%	15%	8%	3%	4%	10%	0%	20%	25%	0%	0%	0%	
	Bilateral	50%	0%	23%	31%	45%	22%	15%	13%	40%	25%	0%	0%	0%	
Location of headache, last 30 days	None	25%	33%	23%	15%	18%	22%	50%	56%	60%	0%	67%	0%	0%	0.056
	temporal	75%	33%	69%	54%	58%	43%	45%	38%	20%	100%	33%	100%	0%	
	Other	0%	33%	8%	31%	24%	35%	5%	6%	20%	0%	0%	0%	100%	
Location of side headache, last 30 days	None	25%	33%	23%	15%	21%	26%	45%	56%	60%	0%	67%	0%	0%	0.06
	Unilateral	25%	33%	46%	8%	18%	9%	25%	13%	0%	50%	0%	100%	0%	
	Bilateral	50%	33%	31%	77%	61%	65%	30%	31%	40%	50%	33%	0%	100%	
Opening pattern	Straight	25%	67%	38%	23%	45%	30%	45%	38%	20%	25%	0%	100%	100%	0.607
	Corrected	50%	0%	54%	62%	52%	61%	55%	56%	80%	75%	100%	0%	0%	
	Uncorrected	25%	33%	8%	15%	3%	9%	0%	6%	0%	0%	0%	0%	0%	
Pain disorders	None	75%	67%	69%	54%	70%	61%	60%	44%	100%	25%	67%	100%	0%	0.466
	Yes	25%	33%	31%	46%	30%	39%	40%	56%	0%	75%	33%	0%	100%	
Myalgia	None	75%	67%	62%	54%	61%	74%	70%	69%	60%	100%	67%	0%	100%	0.858
	Yes	25%	33%	38%	46%	39%	26%	30%	31%	40%	0%	33%	100%	0%	
Myofascial pain with referral	None	50%	67%	85%	100%	79%	87%	85%	100%	60%	75%	100%	100%	100%	0.277
	Yes	50%	33%	15%	0%	21%	13%	15%	0%	40%	25%	0%	0%	0%	
Arthralgia	None	75%	100%	69%	85%	88%	87%	90%	88%	80%	100%	67%	100%	100%	0.435
	Unilateral	0%	0%	31%	15%	3%	13%	10%	6%	0%	0%	33%	0%	0%	
	Bilateral	25%	0%	0%	0%	9%	0%	0%	6%	20%	0%	0%	0%	0%	
Headache attributed to TMD	None	25%	100%	54%	92%	79%	83%	70%	81%	80%	100%	67%	0%	100%	0.103
	Yes	75%	0%	46%	8%	21%	17%	30%	19%	20%	0%	33%	100%	0%	

The consumption of coffee, represented by the number of cups consumed, showed no statistically significant correlation with any of the investigated TMD-related variables (Table 3). This was demonstrated by the Pearson correlation values ranging from -0.046 to 0.095 and significance (two-tailed) values all above the 0.05 threshold, specifically between 0.266 and 0.882 across all variables. When looking at the location of pain in the last 30 days, a strong and statistically significant correlation was observed with the location of side pain (r = 0.698, p < 0.001), indicating that the site of pain was closely related to the side on which the pain was experienced. A moderate correlation was found between the location of pain and the occurrence of headaches (r = 0.242, p = 0.004) and side headaches (r = 0.205, p = 0.015) in the last 30 days, suggesting that general pain location is associated with the presence of headaches. The location of side pain in the last 30 days was highly correlated with the location of headache (r = 0.305, p < 0.001) and the location of side headache (r = 0.329, p < 0.001), indicating a strong relationship between side pain and headaches on the same side. Furthermore, a very strong correlation was found between the location of the headache and the location of the side headache (r = 0.796, p < 0.001), suggesting that when participants experienced headaches, they were likely to also experience side-specific headaches. In terms of opening pattern, none of the correlations with the number of cups of coffee and other TMD-related variables reached statistical significance, indicating no clear relationship between these factors. Pain disorders showed a strong negative correlation with myalgia (r = -0.530, p < 0.001), myofascial pain with referral (r = -0.331, p < 0.001), and arthralgia (r = -0.301, p < 0.001), suggesting that as the presence of pain disorders increased, the incidence of these specific types of pain decreased. Additionally, there was a strong negative correlation between pain disorders and headaches attributed to TMD (r = -0.438, p < 0.001). Myalgia and myofascial pain with referral were strongly negatively correlated (r = -0.302, p < 0.001), indicating a tendency for these conditions to co-occur less frequently than expected by chance. Moreover, a strong positive correlation was observed between myofascial pain with referral and headache attributed to TMD (r = 0.473, p < 0.001), as well as between arthralgia and headache attributed to TMD (r = 0.444, p < 0.001), which indicates that participants with myofascial pain and arthralgia were more likely to also report TMD-related headaches.

**Table 3:** Correlation between cups of coffee and other variables * represents a statistically significant correlation at the 0.05 significance level, i.e., p-value less than 0.05, and ** represents a statistically significant correlation at a more stringent significance level of 0.01, i.e., p-value less than 0.01. TMD: temporomandibular disorder.

Variable analyzed	Statistical test	Cups of coffee	Location of pain, last 30 days	Location of side pain, last 30 days	Location of headache, last 30 days	Location of side headache, last 30 days	Opening pattern	Pain disorders	Myalgia	Myofascial pain with referral	Arthralgia	Headache attributed to TMD
Cups of coffee	Pearson correlation	1	.095	.068	.014	.045	-.034	.044	.040	-.044	.013	-.046
	Sig. (2-tailed)	-	.266	.429	.871	.601	.691	.604	.644	.604	.882	.591
	N	139	139	139	139	139	139	139	139	139	139	139
Location of pain, last 30 days	Pearson correlation	.095	1	.698^**^	.242^**^	.205^*^	.095	-.077	-.042	.090	.171^*^	.039
	Sig. (2-tailed)	.266	-	.000	.004	.015	.267	.368	.623	.291	.045	.647
	N	139	139	139	139	139	139	139	139	139	139	139
Location of side pain, last 30 days	Pearson correlation	.068	.698^**^	1	.305^**^	.329^**^	.000	-.085	-.094	.236^**^	.122	.213^*^
	Sig. (2-tailed)	.429	.000	-	.000	.000	.998	.321	.273	.005	.152	.012
	N	139	139	139	139	139	139	139	139	139	139	139
Location of headache, last 30 days	Pearson correlation	.014	.242^**^	.305^**^	1	.796^**^	.108	-.148	-.013	.158	.085	.191^*^
	Sig. (2-tailed)	.871	.004	.000	-	.000	.207	.082	.879	.064	.318	.024
	N	139	139	139	139	139	139	139	139	139	139	139
Location of side headache, last 30 days	Pearson correlation	.045	.205^*^	.329^**^	.796^**^	1	.071	-.150	-.014	.253^**^	.086	.228^**^
	Sig. (2-tailed)	.601	.015	.000	.000	-	.409	.077	.873	.003	.313	.007
	N	139	139	139	139	139	139	139	139	139	139	139
Opening pattern	Pearson correlation	-.034	.095	.000	.108	.071	1	-.041	-.038	-.017	.102	.035
	Sig. (2-tailed)	.691	.267	.998	.207	.409	-	.635	.658	.840	.234	.683
	N	139	139	139	139	139	139	139	139	139	139	139
Pain disorders	Pearson correlation	.044	-.077	-.085	-.148	-.150	-.041	1	-.530^**^	-.331^**^	-.301^**^	-.438^**^
	Sig. (2-tailed)	.604	.368	.321	.082	.077	.635	-	.000	.000	.000	.000
	N	139	139	139	139	139	139	139	139	139	139	139
Myalgia	Pearson correlation	.040	-.042	-.094	-.013	-.014	-.038	-.530^**^	1	-.302^**^	-.056	-.041
	Sig. (2-tailed)	.644	.623	.273	.879	.873	.658	.000	-	.000	.514	.628
	N	139	139	139	139	139	139	139	139	139	139	139
Myofascial pain with referral	Pearson correlation	-.044	.090	.236^**^	.158	.253^**^	-.017	-.331^**^	-.302^**^	1	.209^*^	.473^**^
	Sig. (2-tailed)	.604	.291	.005	.064	.003	.840	.000	.000	-	.014	.000
	N	139	139	139	139	139	139	139	139	139	139	139
Arthralgia	Pearson correlation	.013	.171^*^	.122	.085	.086	.102	-.301^**^	-.056	.209^*^	1	.444^**^
	Sig. (2-tailed)	.882	.045	.152	.318	.313	.234	.000	.514	.014	-	.000
	N	139	139	139	139	139	139	139	139	139	139	139
Headache attributed to TMD	Pearson correlation	-.046	.039	.213^*^	.191^*^	.228^**^	.035	-.438^**^	-.041	.473^**^	.444^**^	1
	Sig. (2-tailed)	.591	.647	.012	.024	.007	.683	.000	.628	.000	.000	-
	N	139	139	139	139	139	139	139	139	139	139	139

As elucidated through Table 4, the number of sleep hours showed a small but statistically significant negative correlation with the location of pain experienced in the last 30 days; as sleep hours increased, the frequency or intensity of pain in the last 30 days slightly decreased (r = - 0.193, p = 0.023). However, correlations between sleep hours and the location of side pain, headache, and side headache in the last 30 days were also negative but not statistically significant (p > 0.05). The location of pain in the last 30 days had a very strong positive correlation with the location of side pain in the last 30 days (r = 0.698, p < 0.001). It also showed a small positive correlation with the location of headache (r = 0.242, p = 0.004) and the location of side headache (r = 0.205, p = 0.015) in the last 30 days. There was a weak positive correlation with arthralgia (r = 0.171, p = 0.045) but no significant correlation with sleep hours, opening pattern, pain disorders, myalgia, or myofascial pain with referral. The location of side pain in the last 30 days had a moderately positive correlation with the location of headache (r = 0.305, p < 0.001) and the location of side headache (r = 0.329, p < 0.001) in the last 30 days. Additionally, there was a small positive correlation with the headache attributed to TMD (r = 0.213, p = 0.012). No significant correlation was found with sleep hours, opening pattern, pain disorders, myalgia, or arthralgia. The location of the headache in the last 30 days showed a very strong positive correlation with the location of side headache (r = 0.796, p < 0.001) and a small positive correlation with the headache attributed to TMD (r = 0.191, p = 0.024). No significant correlation was observed with sleep hours, opening pattern, pain disorders, myalgia, myofascial pain with referral, or arthralgia. The location of side headaches in the last 30 days demonstrated a moderate positive correlation with myofascial pain with referral (r = 0.253, p = 0.003) and a small positive correlation with the headache attributed to TMD (r = 0.228, p = 0.007). No significant correlations were found with sleep hours, opening pattern, pain disorders, myalgia, or arthralgia. The opening pattern did not show any significant correlations with sleep hours, location of pain, location of side pain, location of headache, location of side headache, pain disorders, myalgia, myofascial pain with referral, arthralgia, or headache attributed to TMD. Pain disorders had a strong negative correlation with myalgia (r = -0.530, p < 0.001), myofascial pain with referral (r = -0.331, p < 0.001), arthralgia (r = -0.301, p < 0.001), and headaches attributed to TMD (r = -0.438, p < 0.001). There were no significant correlations with sleep hours, location of pain, location of side pain, location of headache, location of side headache, or opening pattern. Myalgia and myofascial pain with referral had a strong negative correlation with each other (r = -0.302, p < 0.001). Myalgia showed no significant correlations with sleep hours, location of pain, location of side pain, location of headache, location of side headache, opening pattern, arthralgia, or headache attributed to TMD. Myofascial pain with referral had a moderate positive correlation with headache attributed to TMD (r = 0.473, p < 0.001) and a small positive correlation with arthralgia (r = 0.209, p = 0.014). No significant correlations were found with sleep hours, location of pain, location of side pain, location of headache, location of side headache, opening pattern, or myalgia. Arthralgia showed a moderately positive correlation with headache attributed to TMD (r = 0.444, p < 0.001). No significant correlations were noted with sleep hours, location of pain, location of side pain, location of headache, location of side headache, opening pattern, or myalgia. Headache attributed to TMD had no significant correlations with sleep hours, location of pain, location of side pain, location of headache, location of side headache, opening pattern, or myalgia.

**Table 4:** Correlation between sleep hours and other variables * represents a statistically significant correlation at the 0.05 significance level, i.e., p-value less than 0.05, and ** represents a statistically significant correlation at a more stringent significance level of 0.01, i.e., p-value less than 0.01. TMD: temporomandibular disorder.

Variable analyzed	Statistical test	Sleep hours	Location of pain, last 30 days	Location of side pain, last 30 days	Location of headache, last 30 days	Location of side headache, last 30 days	Opening pattern	Pain disorders	Myalgia	Myofascial pain with referral	Arthralgia	Headache attributed to TMD
Sleep hours	Pearson correlation	1	-.193^*^	-.156	-.138	-.132	-.073	.097	-.066	-.100	-.069	-.087
	Sig. (2-tailed)	-	.023	.067	.105	.121	.390	.256	.437	.242	.419	.306
	N	139	139	139	139	139	139	139	139	139	139	139
Location of pain, last 30 days	Pearson correlation	-.193^*^	1	.698^**^	.242^**^	.205^*^	.095	-.077	-.042	.090	.171^*^	.039
	Sig. (2-tailed)	.023	-	.000	.004	.015	.267	.368	.623	.291	.045	.647
	N	139	139	139	139	139	139	139	139	139	139	139
Location of side pain, last 30 days	Pearson correlation	-.156	.698^**^	1	.305^**^	.329^**^	.000	-.085	-.094	.236^**^	.122	.213^*^
	Sig. (2-tailed)	.067	.000	-	.000	.000	.998	.321	.273	.005	.152	.012
	N	139	139	139	139	139	139	139	139	139	139	139
Location of headache, last 30 days	Pearson correlation	-.138	.242^**^	.305^**^	1	.796^**^	.108	-.148	-.013	.158	.085	.191^*^
	Sig. (2-tailed)	.105	.004	.000	-	.000	.207	.082	.879	.064	.318	.024
	N	139	139	139	139	139	139	139	139	139	139	139
Location of side headache, last 30 days	Pearson correlation	-.132	.205^*^	.329^**^	.796^**^	1	.071	-.150	-.014	.253^**^	.086	.228^**^
	Sig. (2-tailed)	.121	.015	.000	.000	-	.409	.077	.873	.003	.313	.007
	N	139	139	139	139	139	139	139	139	139	139	139
Opening pattern	Pearson correlation	-.073	.095	.000	.108	.071	1	-.041	-.038	-.017	.102	.035
	Sig. (2-tailed)	.390	.267	.998	.207	.409	-	.635	.658	.840	.234	.683
	N	139	139	139	139	139	139	139	139	139	139	139
Pain disorders	Pearson correlation	.097	-.077	-.085	-.148	-.150	-.041	1	-.530^**^	-.331^**^	-.301^**^	-.438^**^
	Sig. (2-tailed)	.256	.368	.321	.082	.077	.635	-	.000	.000	.000	.000
	N	139	139	139	139	139	139	139	139	139	139	139
Myalgia	Pearson correlation	-.066	-.042	-.094	-.013	-.014	-.038	-.530^**^	1	-.302^**^	-.056	-.041
	Sig. (2-tailed)	.437	.623	.273	.879	.873	.658	.000	-	.000	.514	.628
	N	139	139	139	139	139	139	139	139	139	139	139
Myofascial pain with referral	Pearson correlation	-.100	.090	.236^**^	.158	.253^**^	-.017	-.331^**^	-.302^**^	1	.209^*^	.473^**^
	Sig. (2-tailed)	.242	.291	.005	.064	.003	.840	.000	.000	-	.014	.000
	N	139	139	139	139	139	139	139	139	139	139	139
Arthralgia	Pearson correlation	-.069	.171^*^	.122	.085	.086	.102	-.301^**^	-.056	.209^*^	1	.444^**^
	Sig. (2-tailed)	.419	.045	.152	.318	.313	.234	.000	.514	.014	-	.000
	N	139	139	139	139	139	139	139	139	139	139	139
Headache attributed to TMD	Pearson correlation	-.087	.039	.213^*^	.191^*^	.228^**^	.035	-.438^**^	-.041	.473^**^	.444^**^	1
	Sig. (2-tailed)	.306	.647	.012	.024	.007	.683	.000	.628	.000	.000	-
	N	139	139	139	139	139	139	139	139	139	139	139

**Figure 2:**
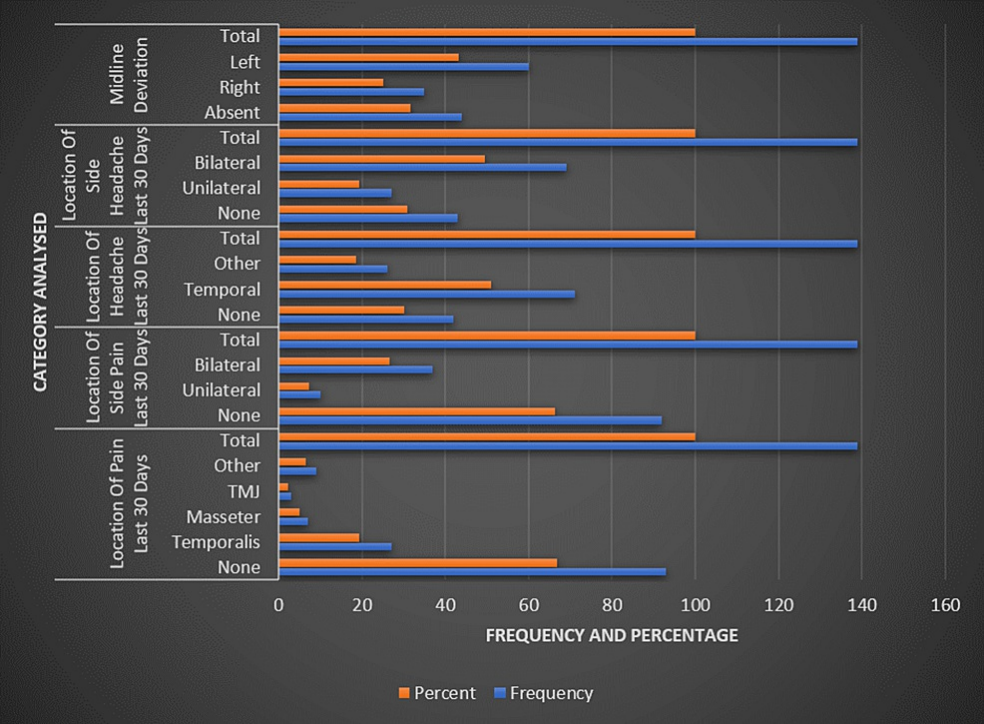
Distribution of jaw function and pain disorders in TMD patients TMD: temporomandibular disorder; TMJ: temporomandibular joint.

The analysis of pain location over the past 30 days revealed that the majority (66.9%) experienced no pain, while 19.4% reported pain in the temporalis muscle, 5% in the masseter muscle, 2.2% in the TMJ, and 6.5% in other areas (Figure 2). Similarly, when examining the side of pain, 66.2% of the sample reported no pain, 7.2% reported unilateral pain, and 26.6% reported bilateral pain. Regarding headaches in the last 30 days, 30.2% of participants had none, 51.1% had temporal headaches, and 18.7% had headaches in other locations. The side of the headache mirrored this, with 30.9% having no headache, 19.4% having unilateral headaches, and 49.6% having bilateral headaches. Midline deviation was not present in 31.7% of the subjects, whereas 25.2% deviated to the right and 43.2% to the left.

**Figure 3:**
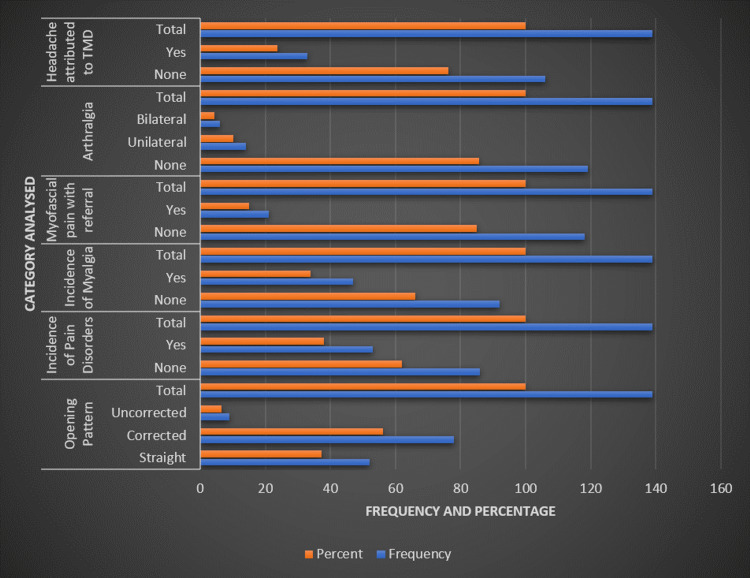
Prevalence of specific TMD-related pain conditions in the study population TMD: temporomandibular disorder.

Figure 3 provides further insights, showing that 37.4% of the sample had a straight opening pattern of the jaw, 56.1% corrected, and 6.5% uncorrected. In terms of pain disorders, 61.9% of participants had no pain disorder, while 38.1% did. Myalgia was absent in 66.2% and present in 33.8% of the subjects. Myofascial pain with referral was not found in 84.9% but was present in 15.1%. Arthralgia was absent in 85.6% of the participants, with 10.1% experiencing unilateral and 4.3% bilateral arthralgia. Headache attributed to TMD was not present in 76.3% of the study population, whereas 23.7% suffered from it.

**Figure 4:**
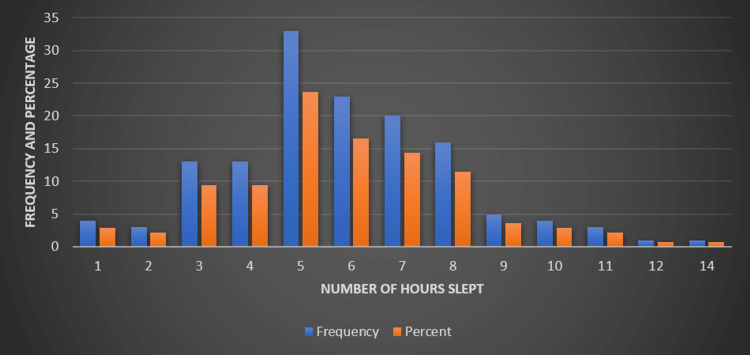
Self-reported hours of sleep among study participants

As evident in Figure 4, it was found that a small number of participants (2.9%) reported sleeping only for one hour, while 2.2% reported sleeping for two hours. Sleep duration increased incrementally, with 9.4% of the participants reporting between three and four hours of sleep. The most common sleep duration was five hours, reported by 23.7% of participants, followed by 16.5% reporting six hours and 14.4% reporting seven hours of sleep. A smaller proportion of participants reported longer sleep durations: 11.5% reported eight hours, 3.6% reported nine hours, and 2.9% reported 10 hours. Very few participants reported sleeping for 11 or 12 hours, each constituting 2.2% and 0.7%, respectively, and a single participant (0.7%) reported sleeping for 14 hours.

**Figure 5:**
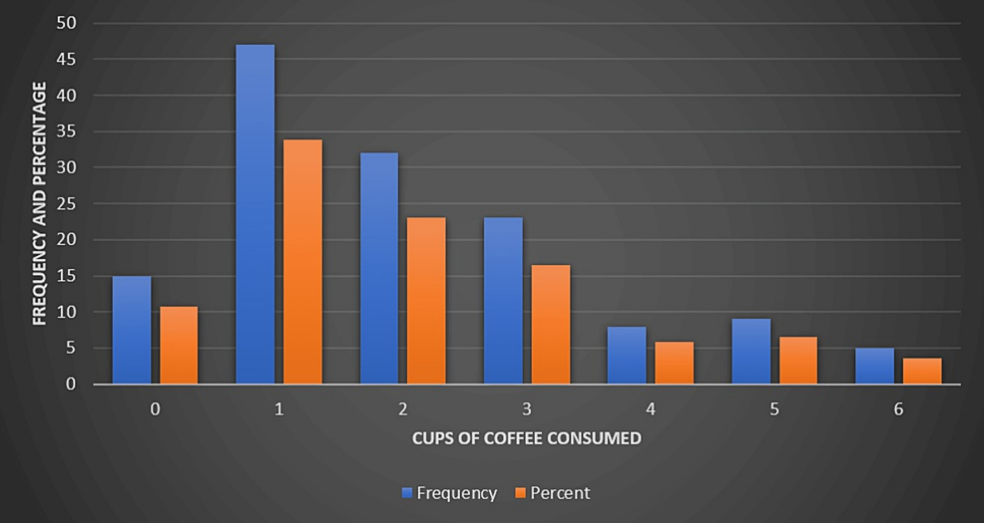
Daily coffee consumption patterns among study participants

Regarding caffeine consumption (Figure 5), the study revealed that 10.8% of participants did not consume any coffee. The largest group of coffee consumers consisted of those who drank one cup daily, representing 33.8% of participants. This was followed by those who consumed two cups (23%) and three cups (16.5%). A smaller percentage of the sample reported higher coffee consumption: 5.8% drank four cups, 6.5% drank five cups, and 3.6% reported consuming six cups of coffee.

## Discussion

The significance of this study lies in several key aspects. A diverse set of parameters, including height, weight, BMI, age, location of pain, side of pain, location of headache, and various pain disorders, were examined, which increased the potential to generalize the findings across different categories of the population. By doing so, it provides a comprehensive view of the multifactorial nature of TMD and its potential correlations with these factors. This comprehensive approach enhances our understanding of the condition and its management. Also, the study's inclusion of both symptomatic and asymptomatic TMD cases is noteworthy. This approach allows for a comparative analysis that can elucidate the differences between these two groups, shedding light on potential risk factors or protective factors related to TMD development. Such information can inform early intervention and prevention strategies. Furthermore, the study's focus on sleep, caffeine intake, and BMI as potential influencing factors on pain pressure thresholds in TMD cases adds a novel dimension to TMD research. Understanding how lifestyle factors, such as sleep duration and dietary habits, may relate to TMD symptoms can guide patient management and lifestyle recommendations. The future implications of this study include the potential for more targeted and personalized interventions for TMD patients. If correlations between sleep, caffeine intake, BMI, and pain pressure thresholds are established, healthcare providers can tailor treatment plans to address these specific factors. Additionally, this research may encourage further investigations into the mechanistic pathways linking these variables to TMD, potentially leading to new therapeutic approaches. TMDs and oral parafunctions are prevalent in modern society. The manual evaluation of myofascial pain is traditionally considered the gold standard for diagnosing muscle disorders [15-17]. However, the variability in applied pressure during palpation has always been a critical limitation. Too much pressure can lead to false positive responses, while insufficient pressure can result in false negatives [18,19]. Algometry addresses this issue by standardizing the pressure applied during assessment, ensuring consistency for both symptomatic and asymptomatic individuals [15-17,20,21]. The use of an algometer is deemed reliable as it allows for uniform application of pressure to the muscles and structures being tested [21]. The present study benefits from the consistent calibration of pressure, which mitigates bias and reduces the incidence of false positives and negatives. In contrast, Liljestrom et al. did not observe an association between sleep quality and TMD among children [22]. Conversely, research by Rafael dos Santos Silva et al. indicated a significant correlation between sleep duration and myofascial pain [23], and Vazquez-Delgado et al. reported poorer sleep quality in patients with myofascial pain [24].

Dodick et al. found that individuals with primary headaches accompanied by sleep disorders also exhibited axis II conditions, such as depression and anxiety, as well as a history of pain medication abuse and fibromyalgia [25]. These conditions independently affected sleep quality. In our study, hours of sleep had a statistically significant negative correlation with the location of pain in the last 30 days (-0.193) and a positive correlation with the location of the headache in the last 30 days (0.205) and with arthralgia (0.171). These results support the hypothesis that reduced sleep duration may increase the risk of a cascade of pain-related issues. Clinically, it is often observed that patients with insomnia and myofascial pain syndrome report an exacerbation of symptoms during the day following poor sleep quality at night [26]. Our results revealed that the majority of pain was localized to the temporalis muscle (n = 27, 19.4%), commonly associated with tension-type headaches (TTH). Additionally, the reported location of headaches in the past month was predominantly in the temporalis region (n = 71, 51.1%). Myalgia and myofascial pain with referral were also noted in 47 (33.8%) and 21 (15.1%) of participants, respectively. These findings align with the notion that limited sleep can initiate and exacerbate pain, particularly in individuals with chronic pain conditions such as myofascial pain syndrome (MPS) [26]. Participants averaged 5.9 hours of sleep and consumed two cups of coffee daily. Interestingly, caffeine intake had a statistically significant impact on pain disorders, myofascial pain with referral, and arthralgia (p-value = 0.000). Despite an average consumption of two cups of coffee, which is below the FDA-recommended four to five cups per day [10], we did not account for the timing of caffeine intake, which could disrupt sleep patterns and aggravate symptoms due to caffeine's half-life of four to six hours. BMI showed a notable correlation only with temporalis pain and headache location. Research on male veterans with a high BMI (>27 kg/m^2^) indicated a significant impact of obesity on pain [27]. A systematic review and a cross-sectional study on obesity and pain highlighted a cyclical relationship where pain leads to sedentary behavior, promoting weight gain, which in turn exacerbates pain due to the pro-inflammatory state associated with a higher BMI [28,29]. In our study, the mean BMI was 26, indicating an overweight but non-obese sample. It has been established that a BMI ≥ 30 is a critical threshold at which BMI significantly influences pain perception and treatment efficacy [27-29]. This is supported in a study by Koçyiğit et al. [30], who found that obesity had a more substantial effect on pain compared to lower BMI categories in females with fibromyalgia when divided into normal weight (BMI = 18.5-24.9), overweight (BMI = 25.0-29.9), and obese (BMI ≥ 30) groups. Despite the significance of the obtained findings, the limitations of the present study are multifaceted and contribute to a nuanced understanding of the findings. Initially, the study design was observational and cross-sectional, which inherently restricted the ability to establish causality between the examined lifestyle factors and PPT outcomes in TMDs. The utilization of a convenience sample may induce selection bias, as the participants were females visiting a single center in Riyadh city, potentially limiting the generalizability of the results to broader populations with different demographic and cultural backgrounds. Furthermore, the age range of the participants was confined to 20-50 years, which may not be reflective of the TMD experience in other age groups, particularly the geriatric population, which could have different lifestyle patterns and pain perceptions. The data collection tools included an operator-designed questionnaire and a symptom questionnaire, which, while tailored to the study's objectives, may not have been previously validated for reliability and consistency across diverse populations. The lack of control over potential confounding variables is another limitation. While the study focused on sleep duration, caffeine intake, and BMI, other lifestyle factors such as stress levels, dietary habits, physical activity, and overall health status were not accounted for and may influence PPT values. The sample size, although adequate for preliminary investigation, may not have the power to detect subtle associations or the full spectrum of variability in PPT across the TMD patient population.

## Conclusions

Our study's findings about the effects of coffee and the significant differences between sleeping hours and PPT are in line with earlier research. Notably, sleep was found to be a significant element that had a considerable impact on TMD patients' PPT values. Although these results highlight the significance of sleep in TMD pain perception, the study was limited by its cross-sectional methodology and emphasis on a particular female subgroup, overlooking the importance of timing caffeine intake before bed. Further research on the relationship between obesity and pain may benefit from a larger sample size of people with higher BMIs. Future studies ought to investigate the causative pathways and take into account a wider range of populations in their longitudinal analyses. This study advances our knowledge of the pain associated with TMDs and raises the possibility of future advancements in diagnosis and treatment approaches.

## References

[1] Chaput JP, Gray CE, Poitras VJ (2017). Systematic review of the relationships between sleep duration and health indicators in the early years (0-4 years). BMC Public Health.

[2] Owens J (2014). Insufficient sleep in adolescents and young adults: an update on causes and consequences. Pediatrics.

[3] Institute of Medicine (2006). Sleep Disorders and Sleep Deprivation: An Unmet Public Health Problem.

[4] Rhim E, Han K, Yun KI (2016). Association between temporomandibular disorders and obesity. J Craniomaxillofac Surg.

[5] Jordani PC, Campi LB, Circeli GZ, Visscher CM, Bigal ME, Gonçalves DA (2017). Obesity as a risk factor for temporomandibular disorders. J Oral Rehabil.

[6] Kim SY, Yoo DM, Byun SH (2021). Association between temporomandibular joint disorder and weight changes: a longitudinal follow-up study using a national health screening cohort. Int J Environ Res Public Health.

[7] Fricton J (2016). Myofascial pain: mechanisms to management. Oral Maxillofac Surg Clin North Am.

[8] Shaefer JR, Khawaja SN, Bavia PF (2018). Sex, gender, and orofacial pain. Dent Clin North Am.

[9] Jafri MS (2014). Mechanisms of myofascial pain. Int Sch Res Notices.

[10] Gerber LH, Sikdar S, Armstrong K (2013). A systematic comparison between subjects with no pain and pain associated with active myofascial trigger points. PM R.

[11] Simons DG (2004). Review of enigmatic MTrPs as a common cause of enigmatic musculoskeletal pain and dysfunction. J Electromyogr Kinesiol.

[12] Kamińska A, Dalewski B, Sobolewska E (2020). The usefulness of the pressure algometer in the diagnosis and treatment of orofacial pain patients: a systematic review. Occup Ther Int.

[13] Schmitter M, Kress B, Leckel M, Henschel V, Ohlmann B, Rammelsberg P (2008). Validity of temporomandibular disorder examination procedures for assessment of temporomandibular joint status. Am J Orthod Dentofacial Orthop.

[14] Farella M, Soneda K, Vilmann A, Thomsen CE, Bakke M (2010). Jaw muscle soreness after tooth-clenching depends on force level. J Dent Res.

[15] Dworkin SF (2010). Research diagnostic criteria for temporomandibular disorders: current status & future relevance. J Oral Rehabil.

[16] Oliveira-Campelo NM, Rubens-Rebelatto J, Martí N-Vallejo FJ, Alburquerque-Sendí N F, Fernández-de-Las-Peñas C (2010). The immediate effects of atlanto-occipital joint manipulation and suboccipital muscle inhibition technique on active mouth opening and pressure pain sensitivity over latent myofascial trigger points in the masticatory muscles. J Orthop Sports Phys Ther.

[17] Crincoli V, Cannavale M, Cazzolla AP, Dioguardi M, Piancino MG, Di Comite M (2021). Temporomandibular disorders and oral features in idiopathic inflammatory myopathies (IIMs) patients: an observational study. Int J Med Sci.

[18] John MT, Dworkin SF, Mancl LA (2005). Reliability of clinical temporomandibular disorder diagnoses. Pain.

[19] Park G, Kim CW, Park SB, Kim MJ, Jang SH (2011). Reliability and usefulness of the pressure pain threshold measurement in patients with myofascial pain. Ann Rehabil Med.

[20] Jensen K, Andersen HØ, Olesen J, Lindblom U (1986). Pressure-pain threshold in human temporal region. Evaluation of a new pressure algometer. Pain.

[21] Fischer AA (1987). Pressure algometry over normal muscles. Standard values, validity and reproducibility of pressure threshold. Pain.

[22] Liljeström MR, Le Bell Y, Anttila P (2005). Headache children with temporomandibular disorders have several types of pain and other symptoms. Cephalalgia.

[23] Silva RS, Conti PCR, Mitrirattanakul S, Merrill R (2011). Muscle pain intensity of patients with myofascial pain with different additional diagnoses. Dental Press J Orthod.

[24] Vazquez-Delgado E, Schmidt JE, Carlson CR, DeLeeuw R, Okeson JP (2004). Psychological and sleep quality differences between chronic daily headache and temporomandibular disorders patients. Cephalalgia.

[25] Dodick DW, Eross EJ, Parish JM, Silber M (2003). Clinical, anatomical, and physiologic relationship between sleep and headache. Headache.

[26] Lin WC, Shen CC, Tsai SJ, Yang AC (2017). Increased risk of myofascial pain syndrome among patients with insomnia. Pain Med.

[27] Higgins DM, Buta E, Heapy AA (2020). The relationship between body mass index and pain intensity among veterans with musculoskeletal disorders: findings from the MSD cohort study. Pain Med.

[28] McVinnie DS (2013). Obesity and pain. Br J Pain.

[29] Pazzianotto-Forti EM, Sgariboldi D, Rasera I Jr, Reid WD (2018). Impact of pain in overweight to morbidly obese women: preliminary findings of a cross-sectional study. Physiotherapy.

[30] Koçyiğit BF, Okyay RA (2018). The relationship between body mass index and pain, disease activity, depression and anxiety in women with fibromyalgia. PeerJ.

